# Both Type I and Type II Interferons Can Activate Antitumor M1 Macrophages When Combined With TLR Stimulation

**DOI:** 10.3389/fimmu.2018.02520

**Published:** 2018-11-02

**Authors:** Elisabeth Müller, Martin Speth, Panagiotis F. Christopoulos, Anna Lunde, Ajna Avdagic, Inger Øynebråten, Alexandre Corthay

**Affiliations:** ^1^Tumor Immunology Lab, Department of Pathology, Rikshospitalet, Oslo University Hospital, University of Oslo, Oslo, Norway; ^2^Department of Biosciences, University of Oslo, Oslo, Norway

**Keywords:** macrophages, toll-like receptors, interferon-γ, interferon-α, interferon-β, cancer, nitric oxide, immunotherapy

## Abstract

Triggering or enhancing antitumor activity of tumor-associated macrophages is an attractive strategy for cancer treatment. We have previously shown that the cytokine interferon-γ (IFN-γ), a type II IFN, could synergize with toll-like receptor (TLR) agonists for induction of antitumor M1 macrophages. However, the toxicity of IFN-γ limits its clinical use. Here, we investigated whether the less toxic type I IFNs, IFN-α, and IFN-β, could potentially replace IFN-γ for induction of antitumor M1 macrophages. We measured *in vitro* the ability of type I and II IFNs to synergize with TLR agonists for transcription of inducible nitric oxide synthase (iNOS) mRNA and secretion of nitric oxide (NO) by mouse bone marrow-derived macrophages (BMDMs). An *in vitro* growth inhibition assay was used to measure both cytotoxic and cytostatic activity of activated macrophages against Lewis lung carcinoma (LLC) cancer cells. We found that both type I and II IFNs could synergize with TLR agonists in inducing macrophage-mediated inhibition of cancer cell growth, which was dependent on NO. The ability of high dose lipopolysaccharide (LPS) to induce tumoricidal activity in macrophages in the absence of IFN-γ was shown to depend on induction of autocrine type I IFNs. Antitumor M1 macrophages could also be generated in the absence of IFN-γ by a combination of two TLR ligands when using the TLR3 agonist poly(I:C) which induces autocrine type I IFNs. Finally, we show that encapsulation of poly(I:C) into nanoparticles improved its potency to induce M1 macrophages up to 100-fold. This study reveals the potential of type I IFNs for activation of antitumor macrophages and indicates new avenues for cancer immunotherapy based on type I IFN signaling, including combination of TLR agonists.

## Introduction

During the last decade, breakthrough therapies aiming to unleash the body's own immune response against cancer have provided significant clinical benefits to patient groups previously faced with limited treatment options (1). It has become clear that not only cancer cells, but also the immune and stromal cells within tumors, can be targeted with great effects. One important type of immune cell in tumors is the macrophage (2, 3). Macrophage accumulation in tumors can be detrimental because cancer cells can exploit macrophages for their ability to promote growth, angiogenesis, and immune suppression (4). Nevertheless, macrophages can also acquire potent antitumor activities upon appropriate stimulation. Such M1 macrophages mediate both direct antitumor activity through killing of cancer cells and inhibition of angiogenesis, as well as indirect effects through stimulation of T cells and induction of Th1 immune responses (5–9).

Several interventions targeting macrophages have shown promising results in mouse models for cancer, including a histone deacetylase inhibitor (10), agonistic anti-CD40 antibodies (9, 11), TGF-β inhibition in combination with toll-like receptor 7 (TLR7) ligation (12), and attenuated Listeria monocytogenes (13). Several TLR agonists are currently in clinical trials (14), but so far no therapeutic strategies which directly induces antitumor macrophages have been approved. The best characterized and most established protocol for inducing antitumor macrophages *in vitro* is based on activation with the TLR4 agonist lipopolysaccharide (LPS), alone or in combination with interferon (IFN)-γ (15, 16). Unfortunately, LPS is highly toxic, and IFN-γ has also shown severe dose-dependent side effects, including influenza-like symptoms, nausea, dizziness, anorexia, depression and leukopenia (17, 18). We have previously shown that LPS can be replaced by other, potentially better tolerated TLR ligands such as the TLR1/2 agonist Pam3CSK4 (a lipopeptide that mimics the acylated amino terminus of bacterial lipoproteins), and the TLR7 agonist CL264 (a 9-benzyl-8 hydroxyadenine derivative) for induction of an antitumor macrophage phenotype *in vitro* (19). Both Pam3CSK4 and CL264 were able to synergize with IFN-γ to induce antitumor M1 macrophages, but, unlike LPS, had no effect alone (19).

Combinations of multiple TLR agonists have synergistic effects on the production of proinflammatory cytokines and nitric oxide (NO) by macrophages *in vitro* (20, 21) and on antitumor activity of the immune system *in vivo* (22). All TLRs (except TLR3) signal through the adapter protein MyD88 (myeloid differentiation primary response 88), leading to activation of nuclear factor-κB (NF-κB). A second, MyD88-independent signaling pathway, which results in the induction of type I IFNs, depends on the TRIF adapter molecule (TIR-domain-containing adapter-inducing IFN-β). The TRIF pathway is activated by LPS through TLR4 or poly(I:C) through TLR3 (23–26). We have recently shown that poly(I:C) encapsulated in nanoparticles strongly synergizes with the TLR2 agonist bacille Calmette-Guérin (BCG) in inducing cytokine and NO production in mouse bone-marrow derived macrophages (BMDM) via TRIF-mediated autocrine type I IFN signaling (21). Autocrine signaling through IFN-α/β has also been shown to be crucial for the expression of inducible NO synthase (iNOS) and NO production in response to LPS (27). Expression of iNOS is a well-established marker for mouse proinflammatory M1 macrophages, and NO production is required for macrophage-mediated inhibition of cancer cell growth *in vitro* (19). Therefore, type I IFNs emerge as an attractive mediator for inducing antitumor macrophages.

In this study, we found that autocrine production of type I IFNs was important for the ability of LPS to induce antitumor macrophages in the absence of IFN-γ. We further observed that both recombinant and endogenously produced type I IFNs could synergize with Pam3CSK4 for induction of antitumor macrophages in a similar fashion as IFN-γ. Finally, we could show that macrophage antitumor activity is ~100-fold more efficiently induced in Pam3CSK4/poly(I:C) co-treated macrophages by using poly(I:C)-encapsulated nanoparticles [poly(I:C)-NP] instead of soluble poly(I:C). Our data reveal the potential of type I IFNs in the activation of antitumor macrophages and suggest a potential strategy for macrophage-targeted immunotherapy utilizing combinations of TLR agonists and nanoparticle technology.

## Methods

### Mice

C57BL/6NRj mice were purchased from Janvier Labs (Le Genest-Saint-Isle, France) and bred at the Department of Comparative Medicine, Oslo University Hospital, Rikshospitalet (Oslo, Norway) in specific pathogen free (SPF) conditions. Bones from mice deficient in the IFN alpha/beta receptor 1 (*Ifnar1*^−/−^) were obtained from the Helmholtz Centre for Infection Research, Braunschweig, Germany, and the TWINCORE, Centre for Experimental and Clinical Infection Research, Hannover, Germany (28, 29).

### Cell lines

Lewis lung carcinoma (LLC) is a cell line originating from a spontaneous lung carcinoma in a C57BL/6 mouse and was obtained from CLS Cell Lines Service (Eppelheim, Germany) (30). L929 is a fibroblast-like cell line originating from connective tissue of a C3H/An mouse and was obtained from ATCC (Manassas, VA, USA) (31). Both cell lines were negative for mycoplasma infection as tested by use of MycoSensor PCR Assay kit (#302109, Agilent, Santa Clara, CA, USA).

### TLR agonists and cytokines

The following TLR agonists were used: TLR1/TLR2 agonist Pam3CSK4 (#tlrl-pms, InvivoGen, San Diego, CA, USA); TLR3 agonist polyinosinic:polycytidylic acid [poly(I:C)] of high molecular weight type (#tlrl-pic, InvivoGen); TLR4 agonist LPS from *E. coli* (#L4391, Sigma-Aldrich, St. Louis, MO, USA); and TLR7 agonist CL264 (#tlrl-c264e-5, InvivoGen). The TLR agonists were used alone or in combination with mouse recombinant IFN-γ (#315-05, Peprotech, Rocky Hill, NJ, USA), mouse recombinant IFN-β (#8234-MB, R&D Systems Inc., Minneapolis, MN USA), or mouse recombinant IFN-α type A (#12100-1, PBL Assay Sciences, Piscataway, NJ, USA).

### Production of poly(I:C)-encapsulating nanoparticles [poly(I:C)-NPs]

Poly(I:C)-encapsulating nanoparticles were produced as described previously (21). Briefly, equal volumes of 1 mg/mL of low molecular weight poly(I:C) (#tlrl-picw, InvivoGen) in 0.9% NaCl and 2 mg/mL of chitosan (KiOmedine-CsU, #740063, Sigma-Aldrich) dissolved in MilliQ H_2_O were mixed under stirring at room temperature. Spontaneously formed poly(I:C)-NPs were then collected by centrifugation at 10,000 g for 20 min on a glycerol bed, before resuspension in 0.9% NaCl and dissociation by water bath sonication for 10 min. The content of poly(I:C) encapsulated in poly(I:C)-NPs was determined indirectly by quantification of the amount of non-encapsulated (free) poly(I:C) in the supernatant after the centrifugation step. Poly(I:C) was quantified by measuring the absorbance at 260 nm using a NanoDrop spectrophotometer (Thermo Fisher Scientific, Waltham, MA, USA).

### Generation of bone marrow-derived macrophages (BMDMs)

Mouse macrophages were differentiated from bone marrow cells according to an established protocol using L929 cell-conditioned medium as source of macrophage colony-stimulating factor (M-CSF) (32). The L929 cell-conditioned medium was produced as follows: Confluent L929 cells were cultured in RPMI 1640 medium (#61870044, Thermo Fisher Scientific) containing 10% fetal bovine serum (FBS, #BCHRS0405, Biochrom GmbH, Berlin, Germany) for 10 days before the medium was centrifuged, filtered and stored at −20°C until use. Bone marrow from the femurs and tibiae of the hind legs of 8-12 weeks old male and female C57BL/6NRj mice was flushed with RPMI 1640 medium containing 10% FBS under sterile conditions, and passed through a 70-μm cell strainer (#CLS431751-50EA, Sigma-Aldrich). Red blood cells (RBC) were removed by incubation in RBC lysis buffer (150 mM NH_4_Cl, 10 mM KHCO_3_, and 0.1 mM Na_2_ EDTA) for 10 min at room temperature. After centrifugation and washing, the remaining cells were cultured in 10 cm non-tissue culture treated dishes (#734-2359, VWR, Radnos, PA, USA) in RPMI 1640 medium containing 30% L929-cell-conditioned medium. Bone marrow cells were cultured for 5 days, after which non-adherent cells were washed off using phosphate buffered saline (PBS, #D8662, Sigma-Aldrich) and the adherent macrophages were cultured for an additional day. Differentiated BMDMs were detached by incubation in PBS without CaCl_2_ and MgCl_2_ (#D8537, Sigma-Aldrich) for 30 min at 4°C and by repeated gentle flushing of the dish, before the BMDMs were collected by centrifugation. BMDMs were then frozen in FBS with 10% dimethyl sulfoxide (DMSO, #0231-500 ml, VWR) and stored at −150°C for future experiments. The purity of the cells was ≥99% as routinely analyzed by flow cytometry using the macrophage markers CD11b (clone M1/70, #101219, BioLegend, San Diego, CA, USA) and F4/80 (clone BM8, #123116, BioLegend).

### Tumor cell growth inhibition assay

Frozen aliquots of BMDMs were thawed and cultured for 3 days in non-tissue culture treated dishes (#734-2359, VWR) in RPMI 1640 medium supplemented with 10% FBS (Biochrom) and 10% L929-cell conditioned medium. Next, BMDMs were harvested by scraping and incubated for 2 h at 37°C with 10 mg/mL mitomycin C (#M4287, Sigma-Aldrich) to block eventual proliferation of BMDMs. After washing twice with PBS, BMDMs were seeded in flat bottom 96 well plates (#734-1793, Costar, Washington DC, USA) at density 6 × 10^4^ cells/well in 200 μL RPMI 1640 medium supplemented with 10% FBS (Biochrom) and 10% L929-cell conditioned medium. After 24 h of incubation, the culture medium was replaced with medium containing different combinations of TLR agonists and cytokines and the cells were stimulated for 24 h. Then, half of the cell supernatants (100 μL) were removed and used for quantification of ${\text{NO}}_{2}^{-}$ and cytokines. Target LLC tumor cells were added to each well (3,000 cells in 100 μL), resulting in 20:1 ratio of effector to target cells. LLC cells were also added to wells without BMDMs and were either left untreated or treated with the same concentrations of TLR agonists and cytokines as the wells with co-cultured BMDMs and LLC cells. After 24 h of culturing, cells were pulsed with [^3^H]thymidine (#MT6032, Hartmann Analytic, Braunschweig, Germany) and harvested 18 h later by a freeze and thaw cycle. Quantification of radiolabeled DNA was performed on a 1450 MicroBeta Trilux Microplate Scintillation counter (Perkin Elmer, Waltham, MA, USA) as counts per minutes (cpm). For each condition, triplicate wells were analyzed.

### Quantification of nitrite (${\text{NO}}_{2}^{-}$) by the griess test

Supernatants of BMDM cultures were centrifuged at 410 g for 5 min to remove detached cells and immediately assayed for nitrite (${\text{NO}}_{2}^{-}$) as a measure for macrophage NO production. Fifty microliters of supernatant was added to 50 μL of a mixture of 1% sulphanilamide (#S9251, Sigma-Aldrich) and 5% phosphoric acid (#W290017, Sigma-Aldrich) in MilliQ water (Griess reagent A) and incubated in the dark at room temperature for 10 min. Finally, 50 μL of 0.1% N-(1-napthyl) ethylenediamine (#N9125, Sigma-Aldrich) in MilliQ water (Griess reagent B) was added and the absorbance at 540 nm was measured using a microplate reader (BioTek Instruments, Winooski, VT, USA). For each condition, triplicate wells were analyzed and a serial dilution of NaNO_2_ (#67398, Sigma-Aldrich) was performed to create a standard curve of ${\text{NO}}_{2}^{-}$ in the range of 1.56 to 100 μM.

### Inhibition of iNOS

N-[[3-(aminomethyl)phenyl]methyl]-ethanimidamide dihydrochloride (1400 w, #1415, Tocris/Bio-Techne, MN, USA) is a specific inhibitor of iNOS (33) and was used to block NO production by activated BMDMs.

### Cytokine quantification by luminex technology

BMDMs (6 × 10^4^ cells/well) were cultured in 200 μL RPMI 1640 supplemented with 10% FBS (Biochrom) and 10% L929-cell conditioned medium and activated for 24 h with TLR agonists with or without IFN-γ. Supernatants were collected and centrifuged at 410 g for 5 min to remove cells and stored at −80°C for maximum 1 week. The concentrations of IFN-α and IFN-β were determined by a multiplex Luminex assay (#EPX020-22187-901, ProcartaPlex, ThermoFischer Scientific) according to the manufacturer's instructions. The detection limit was 4 pg/mL for IFN-α and 2 pg/mL for IFN-β. Samples were analyzed in duplicates, using a Bio-Plex MAGPIX Multiplex Reader and Bio-Plex Manager 6.1 software (Bio-Rad Laboratories).

### Cell viability/proliferation assay

LLC cells were seeded in 96 well plates at 10,000 cells/well in 200 μL RPMI medium without phenol red supplemented with 10% FBS. Recombinant IFN-β was added to the cells to achieve final concentrations ranging from 0.1 to 20 ng/mL. After 48 h of incubation, LLC cell growth was analyzed in triplicate wells per condition using the Cell Counting Kit 8 (CCK-8) (#96992, Sigma-Aldrich) according to the manufacturer's instructions. The CCK-8 assay is based on the formation of an orange formazan product from colorless WST-8 reagent by cellular dehydrogenases, which is strongly correlated with the number of cells. The absorbance was measured at 450 nm using a microplate reader (Perkin Elmer).

### Determination of iNOS mRNA levels by real-time quantitative PCR

BMDMs were seeded in 12 well plates (#CLS3513, Sigma-Aldrich) at a density of 4.5 × 10^5^ cells/well in 1 ml/well RPMI 1640 supplemented with 10% FBS and treated with either IFN-γ (40 ng/mL), IFN-β (40 ng/mL), poly(I:C) (100 μg/mL), LPS (1 or 1,000 ng/mL), Pam3CSK4 (100 ng/mL) or combinations thereof for 24 h. Total RNA was extracted using 300 μL/well TRI-Reagent (#T9424-100ML, Merck KGaA, Darmstadt, Germany) and Direct-zol RNA minipreps (#R2072, Zymo Research, Irvine, CA, USA) according to manufacturer's instructions. In total, 250 ng RNA of each sample were reverse transcribed to cDNA using the Primescript RT kit (#RR036A, Takara Bio Inc., Shiga, Japan) according to manufacturer's instructions. Real-time quantitative PCR (RT-qPCR) was performed with 50 ng of the obtained cDNA, using a Kapa SYBR fast qPCR kit (#KK4600, Kapa Biosystems, Wilmington, MA, USA) and 0.2 μM of mRNA specific primers for the mouse gene *Nos2* which encodes iNOS (forward primer: TTCACCCAGTTGTGCATCGACCTA, reverse primer: TCCATGGTCACCTCCAACACAAGA) in selected cycling conditions (95°C for 3 min, 95°C for 3 s, 60°C for 30 s for 40 cycles). All samples were run in duplicates and the final values were averaged. Following melting curve analysis, the relative differences in iNOS mRNA levels were expressed using the -ΔCt values (Ct_reference_-Ct_target_) and with 18s RNA (forward primer: CGCTTCCTTACCTGGTTGAT, reverse primer: GAGCGACCAAAGGAACCATA) as the endogenous control.

### Statistical analysis

Multiple groups were compared by using two-way ANOVA followed by a *post-hoc* Dunnett's and Sidak's test for multiple comparisons. *P* < 0.05 were considered statistically significant (^*^p < 0.05, ^**^p < 0.01, ^***^p < 0.001). Statistical analysis was performed using GraphPad Prism 7.02 software.

## Results

### LPS-mediated activation of antitumor m1 macrophages is associated with autocrine production of type I IFNs

**Figure 1:**
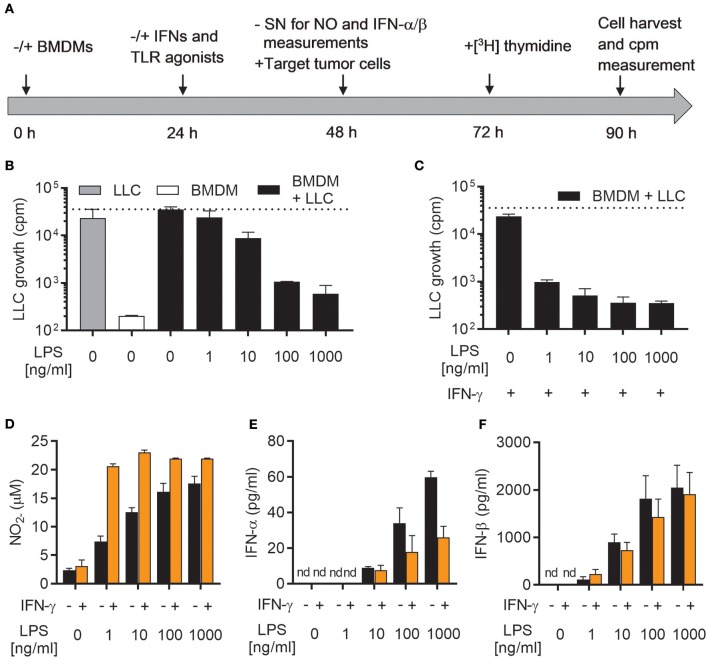
LPS induces growth inhibitory capacity of macrophages and production of both NO and type I IFNs. **(A)** Timeline for the growth inhibition assay used to assess cytotoxic and cytostatic activity of BMDMs toward cancer cells. Mitomycin C-treated BMDMs were seeded out (6 × 10^4^ in 200 μL) and, after 24 h, stimulated with IFNs and/or TLR agonists. At 48 h, half of the cell culture supernatant (SN) was removed for analysis of nitric oxide (NO) and interferon (IFN)-α/β production, before LLC tumor cells (3 × 10^3^) were added to the BMDM cultures, resulting in a 20:1 BMDM effector to LLC target cell ratio. Control wells with LLC cancer cells alone, were treated correspondingly. At 72 h, radiolabeled [^3^H]thymidine was added to all wells, and at 90 h, cells were harvested. Growth of LLC cancer cells was measured by the incorporation of [^3^H]thymidine (counts per minute; cpm). **(B)** Growth inhibition of LLC cells co-cultured with BMDMs, which had been left untreated or stimulated with various concentrations of LPS for 24 h. Cultures of untreated LLC cells alone and mitomycin C-treated BMDMs alone were used as controls. **(C)** Inhibition of growth of LLC cells in co-cultures with BMDMs stimulated for 24 h with 40 ng/mL IFN-γ alone or 40 ng/mL IFN-γ in combination with various concentrations of LPS for 24 h. **(D)** Production of NO by the BMDMs plated for use in analysis of LLC growth inhibition presented in **(B,C)**. The Griess assay was used to measure NO indirectly as nitrite (${\text{NO}}_{2}^{-}$) in the supernatants of BMDMs. **(E,F)** Luminex technology was used to measure the levels of IFN-α **(E)** and IFN-β **(F)** in supernatants from BMDMs plated for analysis of LLC growth inhibition shown in **(B,C)**. The BMDMs were stimulated 24 h with LPS alone or LPS together with IFN-γ. Data are presented as means ± SD of triplicate wells from one representative experiment out of three. nd, not detectable.

We have recently reported that LPS, a classical M1 stimulus, was the only TLR agonist which could activate mouse BMDMs to an antitumor phenotype in the absence of IFN-γ (19). To further investigate this phenomenon, we used a previously established growth inhibition assay (19), in which the growth of cancer cells cultured alone or together with mitomycin C-treated BMDMs was analyzed by measuring the incorporation of radiolabeled thymidine as schematically depicted in Figure 1A. A main advantage of such a growth inhibition assay is that both cytotoxic and cytostatic activities of activated macrophages against cancer cells are measured. BMDMs were left untreated or activated with the indicated factors for 24 h, before NO production and type I IFN secretion were measured in cell culture supernatants (Figure 1A). LLC cancer cells were then added as target cells and co-cultured with BMDMs for another 24 h before radiolabeled [^3^H] thymidine was added for the remaining 18 h of the assay. Finally, [^3^H] thymidine incorporation by LLC cells was analyzed as a measure for cancer cell proliferation (Figure 1A).

As previously reported, stimulation with LPS alone was able to activate BMDMs to inhibit the growth of LLC cancer cells (Figure 1B) (19). This effect was concentration-dependent, and only high concentrations of LPS (100 and 1,000 ng/mL) resulted in efficient LLC cell growth inhibition. Lower concentrations were only partially (10 ng/mL), or not at all (1 ng/mL), able to induce LLC growth inhibition (Figure 1B). Upon co-stimulation with LPS and IFN-γ, BMDMs were able to completely inhibit the growth of LLC cancer cells already at the lowest tested concentration of LPS (1 ng/mL) (Figure 1C). Mirroring the growth inhibition results, production of NO by BMDMs stimulated with LPS alone was concentration-dependent and the lowest concentration of LPS (1 ng/ml) failed to induce substantial levels of NO (Figure 1D). Co-stimulation of BMDMs with LPS/IFN-γ resulted in equally high NO levels for all tested LPS concentrations (1-1,000 ng/ml) (Figure 1D).

A characteristic feature of LPS stimulated leukocytes, such as macrophages and dendritic cells, is the induction of type I IFN production, in particular IFN-α and IFN-β, among other pro-inflammatory cytokines (24). Autocrine activation of the type I IFN signaling pathway has been shown to be essential for LPS-mediated induction of iNOS expression and NO production by macrophages (34, 35). Indeed, stimulation of BMDMs with LPS for 24 h resulted in significant amounts of both IFN-α and IFN-β, which varied with the concentration of LPS (Figures 1E,F). The levels of IFN-α and IFN-β were not further increased by co-stimulation with IFN-γ (Figures 1E,F). In fact, macrophages activated by LPS/IFN-γ appeared to secrete less IFN-α compared to LPS stimulation alone (Figure 1E). Thus, activation of antitumor M1 macrophages by LPS (alone or together with IFN-γ) is associated with autocrine production of the type I IFNs, IFN-α, and IFN-β.

### Autocrine type I IFN signaling is required for lps-induced no production and optimal cancer cell growth inhibition in the absence of IFN-γ

**Figure 2:**
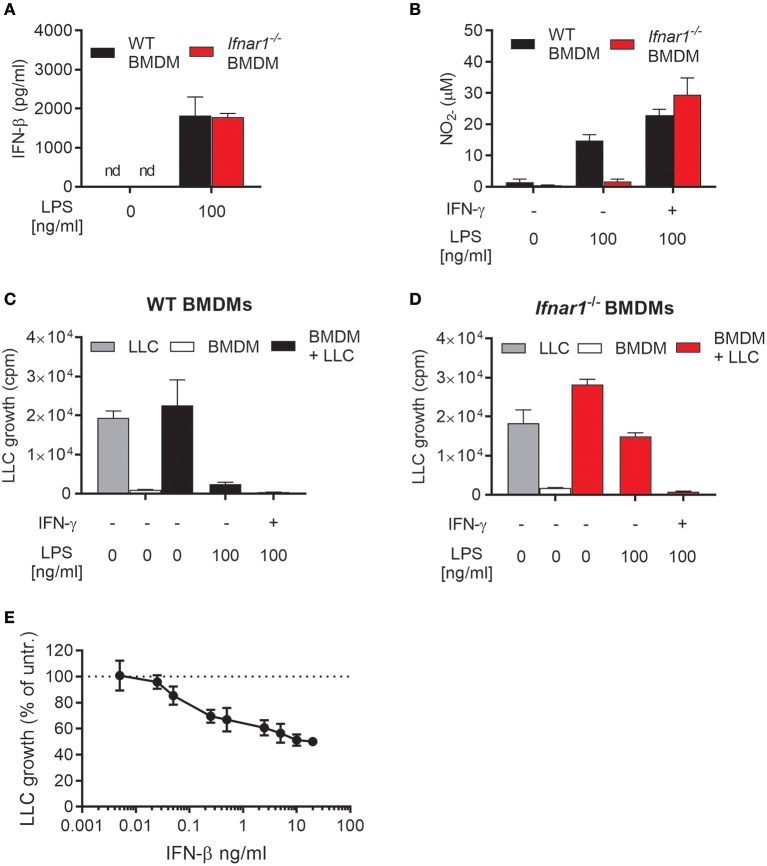
Role of autocrine type I IFNs for NO production and inhibitory activity of macrophages activated by LPS. **(A)** Levels of IFN-β in the supernatant of WT and IFN alpha/beta receptor 1 knockout (*Ifnar1*^−/−^) BMDMs left untreated or treated with 100 ng/mL LPS for 24 h. Data are presented as means ± SD of triplicate wells from a single experiment. **(B)** NO production, measured indirectly as nitrite (${\text{NO}}_{2}^{-}$), by WT and *Ifnar1*^−/−^ BMDMs (6 × 10^4^ in 200 μL) in response to stimulation for 24 h with 100 ng/mL LPS alone or in combination with 40 ng/mL IFN-γ. **(C,D)** Growth inhibition of LLC cells co-cultured with WT **(C)** or *Ifnar1*^−/−^**(D)** BMDMs, which were stimulated as described in **(B)**. Data in **(B–D)** are presented as means ± SD of triplicate wells from one representative experiment out of three. **(E)** Direct effect of exogenous IFN-β on the growth of LLC cells. The proportion of viable cells after 48 h of treatment with different concentrations of IFN-β was determined using the CCK-8 viability assay. Untreated cells were used as control and the results are presented in percentage of the untreated control group. Pooled data from four independent experiments are presented as means ± SD. nd, not detectable.

We investigated the importance of autocrine type I IFN signaling for the induction of an antitumor phenotype in macrophages activated with LPS. For that purpose, we used BMDMs derived from *Ifnar1*^−/−^mice, which lack the common receptor for both IFN-α and IFN-β. WT and *Ifnar1*^−/−^ BMDMs did not differ in their ability to produce IFN-β after treatment with 100 ng/mL LPS for 24 h (Figure 2A). In contrast, *Ifnar1*^−/−^ BMDMs failed to produce NO in response to 100 ng/mL LPS alone (Figure 2B). NO production by *Ifnar1*^−/−^ BMDMs was not reduced compared to WT BMDMs upon co-stimulation with LPS and IFN-γ (Figure 2B). Furthermore, *Ifnar1*^−/−^ BMDMs stimulated with LPS alone were much less able to inhibit LLC cancer cell growth compared to WT BMDMs (Figures 2C,D). When co-stimulated with LPS/IFN-γ, *Ifnar1*^−/−^BMDMs did not differ from WT BMDMs in their ability to completely inhibit LLC cell growth (Figures 2C,D).

*Ifnar1*^−/−^ BMDMs activated with LPS alone retained some inhibitory effect on LLC cells as compared with untreated *Ifnar1*^−/−^ BMDMs (Figure 2D). Notably, *Ifnar1*^−/−^ BMDMs produced normal levels of IFN-β upon LPS stimulation (Figure 2A). It is well-documented that type I IFNs, including IFN-β, can inhibit the growth of cancer cells in a direct manner (36–38). Therefore, we assessed the direct effect of IFN-β on the growth of LLC cells by measuring the LLC cell viability after incubation with different concentrations of recombinant mouse IFN-β for 48 h (Figure 2E). LLC cell growth was inhibited by relatively low concentrations of IFN-β, and a tendency was observed already at 0.05 ng/mL IFN-β. In contrast to IFN-β, a direct effect of IFN-α or IFN-γ on LLC growth was not observed as measured by the thymidine-based growth inhibition assay (Supplementary Figure 1). Thus, the ability of LPS to induce antitumor macrophages in the absence of IFN-γ depends on production of autocrine type I IFNs by the macrophages. Type I IFN signaling in BMDMs is required for LPS-induced NO production and complete inhibition of tumor cell growth. Additionally, LPS-induced IFN-β production is likely to directly contribute to the growth inhibition of LLC cancer cells.

### IFN-β synergizes with TLR agonists for induction of antitumor M1 macrophages

**Figure 3:**
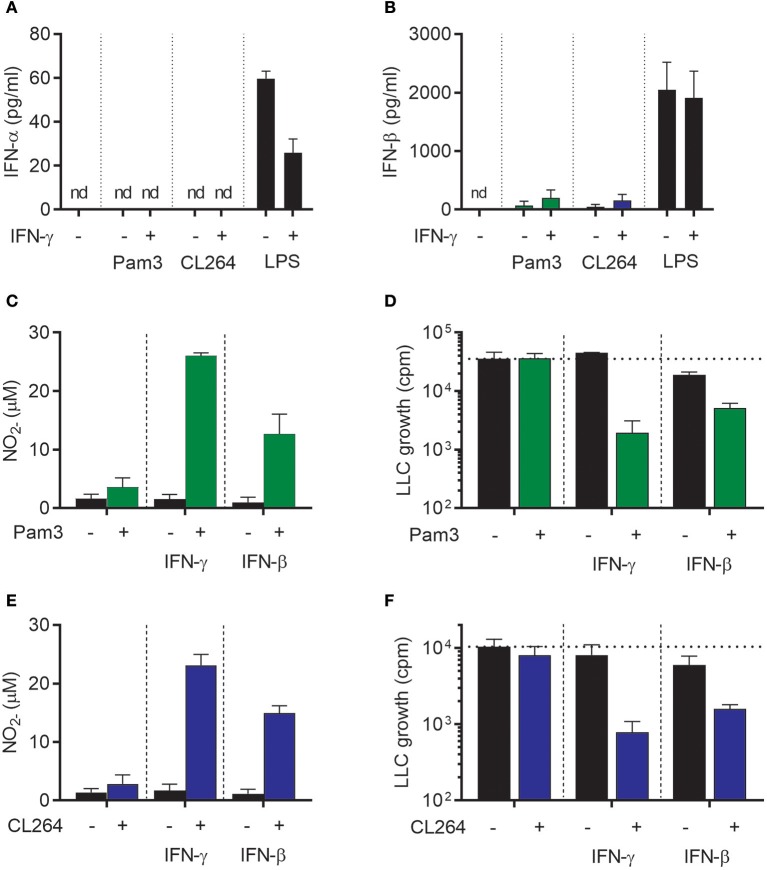
IFN-β synergizes with TLR agonists for activation of antitumor M1 macrophages in a similar fashion as IFN-γ. **(A,B)** Luminex analysis of the levels of IFN-α **(A)** and IFN-β **(B)** produced by BMDMs (6 × 10^4^ in 200 μL) left untreated or stimulated with 100 ng/mL Pam3CSK4, 1 μg/mL CL264, or 1 μg/mL LPS for 24 h in the presence or absence of 40 ng/mL IFN-γ. Note that the results were obtained from the same experiments as the results presented in Figures 1E,F and therefore show the same data for stimulation with 1 μg/ml LPS, which represents the positive control in **(A,B)**. **(C)** NO production, measured indirectly as nitrite (${\text{NO}}_{2}^{-}$), by BMDMs stimulated for 24 h with 100 ng/mL Pam3CSK4, 40 ng/mL IFN-γ, 40 ng/mL IFN-β or the indicated combinations. **(D)** Inhibition of LLC cell growth in BMDM-LLC co-cultures. BMDMs were stimulated as described in **(C)** before addition of LLC cells. **(E)** NO production by BMDMs treated for 24 h with 1 μg/mL CL264, 40 ng/mL IFN-γ, 40 ng/mL IFN-β or the indicated combinations. **(F)** Growth inhibition of LLC cells in co-cultures with BMDMs. The BMDMs were stimulated as described in **(E)** before addition of LLC cells. Data are presented as means ± SD of triplicate wells from one representative experiment out of three. nd, not detectable; Pam3, Pam3CSK4.

We next wanted to examine whether IFN-β could, similarly to IFN-γ, synergize with TLR ligands for induction of antitumor macrophages. The TLR ligands Pam3CSK4 (a TLR1/2 agonist) and CL264 (a TLR7 agonist) were selected because we have previously shown that these TLR agonists cannot alone, but in combination with IFN-γ, induce an antitumor macrophage phenotype (19). First, we tested whether these TLR ligands stimulate macrophages to produce type I IFNs, and measured the levels of IFN-α and IFN-β in the supernatant of BMDMs stimulated for 24 h with Pam3CSK4 or CL264 alone or in combination with IFN-γ. None of the conditions induced detectable levels of IFN-α (Figure 3A). IFN-β could be detected, but at levels ~100-fold lower than upon stimulation with LPS alone or with LPS in combination with IFN-γ (Figure 3B). The data presented in Figures 1E,F, 3A,B are from the same experiment, and the results of stimulation with 1 μg/ml LPS alone or in combination with IFN-γ (Figures 1E,F) are also included in Figures 3A,B but now as positive control.

Stimulation of BMDMs with Pam3CSK4, IFN-γ, or IFN-β for 24 h did not activate BMDMs to produce NO (Figure 3C). However, combined treatment of BMDMs with Pam3CSK4/IFN-γ or with Pam3CSK4/IFN-β induced strong NO production in a synergistic manner (Figure 3C). Furthermore, growth-inhibition of LLC cells was only induced when BMDMs were treated with Pam3CSK4/IFN-γ or Pam3CSK4/IFN-β, whereas Pam3CSK4 alone had no effect (Figure 3D). The effect of CL264 was similar to that of Pam3CSK4 as combined activation with CL264/IFN-γ or with CL264/IFN-β induced strong NO production. Treatment with CL264 alone did not result in NO production (Figure 3E). Co-stimulation of BMDMs with CL264/IFN-γ or CL264/IFN-β was also required for BMDM-mediated growth-inhibition of LLC cells, since CL264 alone had no effect (Figure 3F). In accordance with the data in Figure 2D and Supplementary Figure 1, treatment with exogenous, recombinant IFN-β alone had a limited inhibitory effect on LLC cell growth (Figures 3D,F). Thus, IFN-β synergizes with TLR ligands for induction of NO production and inhibitory activity of macrophages toward cancer cells, in a similar fashion as IFN-γ.

### Both IFN-α and IFN-β synergize with Pam3CSK4 for induction of M1 antitumor macrophages

**Figure 4:**
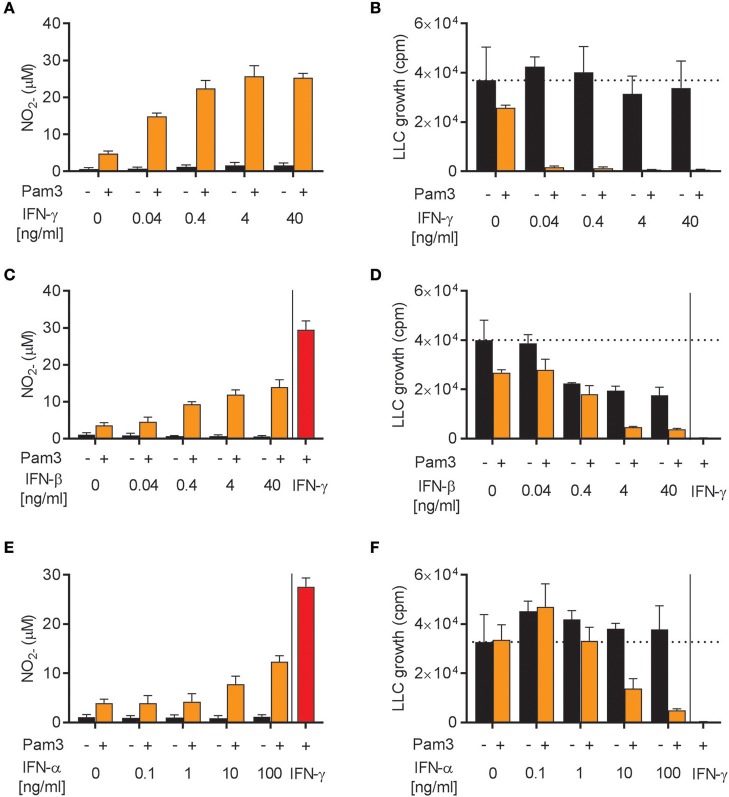
Both IFN-α and IFN-β synergize with Pam3CSK4 in inducing NO production by BMDMs and inhibition of tumor cell growth. **(A)** NO production, measured indirectly as nitrite (${\text{NO}}_{2}^{-}$), by BMDMs (6 × 10^4^ in 200 μL) stimulated for 24 h with the indicated concentrations of IFN-γ in the absence or presence of 100 ng/mL Pam3CSK4. **(B)** BMDMs were stimulated as described in **(A)** before LLC cells (3 × 10^3^) were added to measure inhibition of LLC cell growth in co-cultures. **(C)** NO production by BMDMs stimulated with the indicated concentrations of IFN-β in the absence or presence of 100 ng/mL Pam3CSK4 for 24 h. **(D)** Inhibition of LLC cell growth in co-cultures with BMDMs stimulated as described in **(C)**. **(E)** NO production by BMDMs stimulated with the indicated concentrations of IFN-α in the absence or presence of 100 ng/mL Pam3CSK4 for 24 h. **(F)** Inhibition of LLC cell growth in co-cultures with BMDMs stimulated as described in **(E)**. In **(C–F)** 40 ng/mL IFN-γ was used as positive control. Data are presented as means ± SD of triplicate wells from one representative experiment out of three **(A-F)**. Pam3, Pam3CSK4.

The ability of IFN-α, IFN-β, and IFN-γ to induce antitumor M1 macrophages was investigated in more detail. We stimulated BMDMs with Pam3CSK4 in combination with different concentrations of either IFN-γ, IFN-β, or the other major type I IFN, IFN-α (type A), for 24 h before analyzing NO production and growth inhibition of LLC cancer cells. In accordance with a previous report (39), recombinant IFN-γ synergized with Pam3CSK4 in inducing NO production and BMDM-mediated growth inhibition of cancer cells in a dose-dependent manner starting from the lowest IFN-γ concentration, 0.04 ng/mL (Figures 4A,B). Likewise, recombinant IFN-β as well as IFN-α were able to synergize in a dose-dependent manner with Pam3CSK4 in inducing NO production (Figures 4C,E). Both IFN-β and IFN-α induced BMDM-mediated growth inhibition of cancer cells in combination with Pam3CSK4, although only at relatively high concentrations compared to IFN-γ: at 4 ng/mL and at 100 ng/mL for IFN-β and IFN-α, respectively (Figures 4D,F). We therefore conclude that, similarly to type II IFN, both of the tested type I IFNs can synergize with Pam3CSK4 to induce an antitumor M1 macrophage phenotype.

### NO is required for macrophage-mediated growth inhibition of LLC cells induced by Pam3CSK4 in combination with both type I and type II IFNs

**Figure 5:**
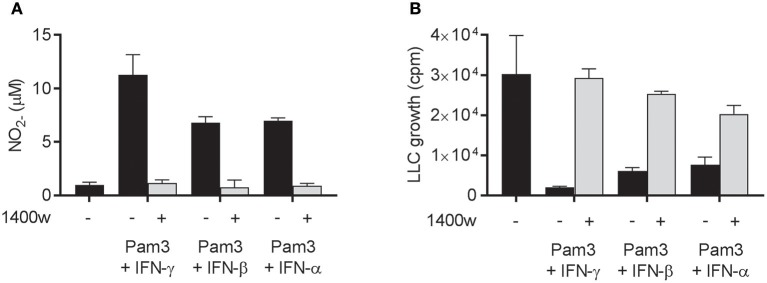
NO is required for macrophage-mediated growth inhibition of LLC cells induced by Pam3CSK4 in combination with both type I and type II IFNs. **(A,B)** BMDMs (6 × 10^4^ in 200 μL) were left untreated or activated for 24 h with Pam3CSK4 (100 ng/mL) and the indicated IFNs (40 ng/mL IFN-γ, 40 ng/mL IFN-β, or 100 ng/mL IFN-α) in the presence or absence of 50 μM of the iNOS inhibitor 1400 w. **(A)** NO production was measured indirectly as nitrite (${\text{NO}}_{2}^{-}$) in the supernatant of untreated or activated BMDMs, and **(B)** inhibition of LLC growth in co-cultures of untreated or activated BMDMs was measured by thymidine incorporation. Data are presented as means ± SD of triplicate wells from one representative experiment out of two. Pam3, Pam3CSK4.

Next, we examined whether the cancer cell growth inhibition induced by Pam3CSK4 in combination with IFN-β or IFN-α was mediated by NO, as it is the case for Pam3CSK4 in combination with IFN-γ (19). NO production was successfully blocked by the iNOS inhibitor 1400 w when macrophages were activated by Pam3CSK4 in combination with any of the IFNs tested (Figure 5A). The presence of 1400 w also abolished the macrophage-mediated inhibition of cancer cell growth induced by Pam3CSK4 and IFNs (Figure 5B). Therefore, we conclude that both type I and type II IFN can be combined with Pam3CSK4 to induce macrophages to inhibit LLC cell growth in an NO-dependent manner.

### Combination of two TLR ligands can be used to activate antitumor M1 macrophages through induction of autocrine type I IFNs

**Figure 6:**
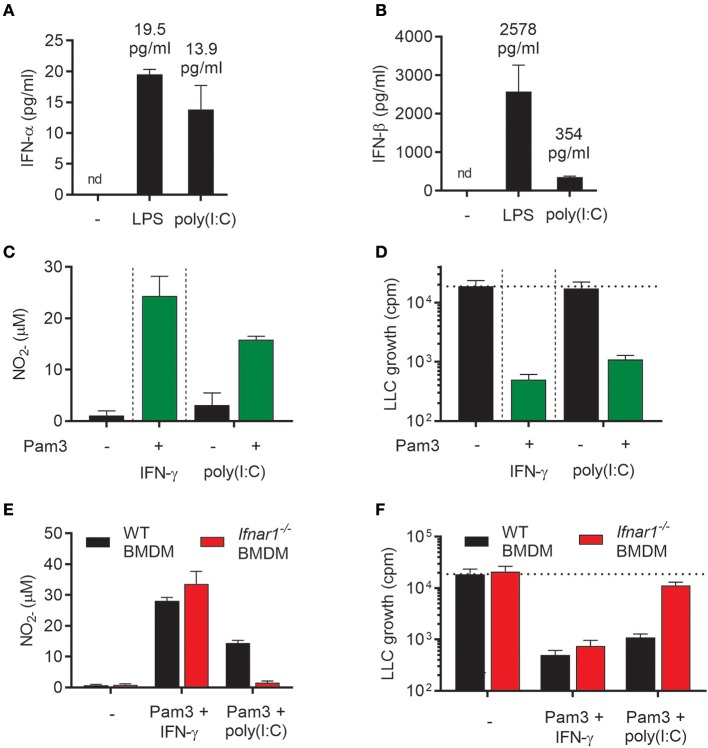
Poly(I:C) synergizes with Pam3CSK4 for activation of antitumor M1 macrophages through induction of type I IFNs. **(A,B)** Production of IFN-α **(A)** and IFN-β **(B)** by BMDMs (6 × 10^4^ in 200 μL) left untreated, activated with 1 μg/mL LPS or 100 μg/mL poly(I:C) for 24 h. **(C)** Production of NO by untreated BMDMs or BMDMs stimulated for 24 h with 100 ng/mL Pam3CSK4 in combination with 40 ng/mL IFN-γ, 100 μg/mL poly(I:C) alone or 100 μg/mL poly(I:C) in combination with 100 ng/mL Pam3CSK4. **(D)** Inhibition of growth of LLC cells (3 × 10^3^ per well) co-cultured with BMDMs (6 × 10^4^), which were stimulated as described in **(C)**. **(E)** NO production by WT and *Ifnar1*^−/−^ BMDMs in response to co-stimulation with Pam3CSK4/IFN-γ or Pam3CSK4/poly(I:C) for 24 h. **(F)** Growth inhibition of LLC cells co-cultured with WT or *Ifnar1*^−/−^ BMDMs treated as described in **(E)**. Data in **(A–F)** are presented as the means ± SD of triplicate wells from one representative experiment out of three. nd, not detectable; Pam3, Pam3CSK4.

In addition to LPS, the TLR3 agonist poly(I:C), which mimics viral double stranded RNA, stimulates macrophages to produce type I IFN via the TRIF signaling pathway (26). We have previously shown that type I IFNs produced upon stimulation with poly(I:C) activated BMDMs in an autocrine manner and that type I IFNs could synergize with TLR ligands, such as the TLR2 agonist Lipomannan and the TLR1/2 agonist Pam3CSK4, in inducing pro-inflammatory macrophages that produce high levels of NO (21). Therefore, we hypothesized that autocrine type I IFN production triggered by poly(I:C) in BMDMs could potentially synergize with Pam3CSK4 in inducing antitumor activity, in a similar manner as recombinant IFN-α or IFN-β. As expected, poly(I:C) induced production of both IFN-α and IFN-β, although the level of IFN-β was much lower than after stimulation with LPS (Figures 6A,B). Stimulation of BMDMs with poly(I:C) alone for 24 h did not result in high NO levels, but the NO production was markedly increased by co-stimulation with poly(I:C) and Pam3CSK4 (Figure 6C). In accordance with these data, we found that BMDMs stimulated with poly(I:C) alone failed to inhibit cancer cell growth, whereas BMDMs stimulated with Pam3CSK4/poly(I:C) inhibited LLC growth to a similar degree as the Pam3CSK4/IFN-γ treated, control BMDMs (Figure 6D).

To study the role of autocrine type I IFN in the induction of antitumor BMDMs by poly(I:C)/Pam3CSK4, *Ifnar1*^−/−^ BMDMs were compared with WT BMDMs in the growth inhibition assay. *Ifnar1*^−/−^ BMDMs co-stimulated with poly(I:C)/Pam3CSK4 completely failed to produce NO (Figure 6E) and showed a strongly reduced ability to inhibit cancer cell growth (Figure 6F). In contrast, neither NO production nor BMDM-mediated growth inhibition was affected when *Ifnar1*^−/−^ BMDMs had been stimulated with Pam3CSK4/IFN-γ (Figures 6E,F). Thus, the combination of two TLR ligands, poly(I:C) and Pam3CSK4, efficiently activated BMDMs to an antitumor phenotype via poly(I:C)-induced autocrine type I IFN signaling.

### Both type I and type II IFNs synergize with TLR agonists for induction of *Nos2* gene expression

**Figure 7:**
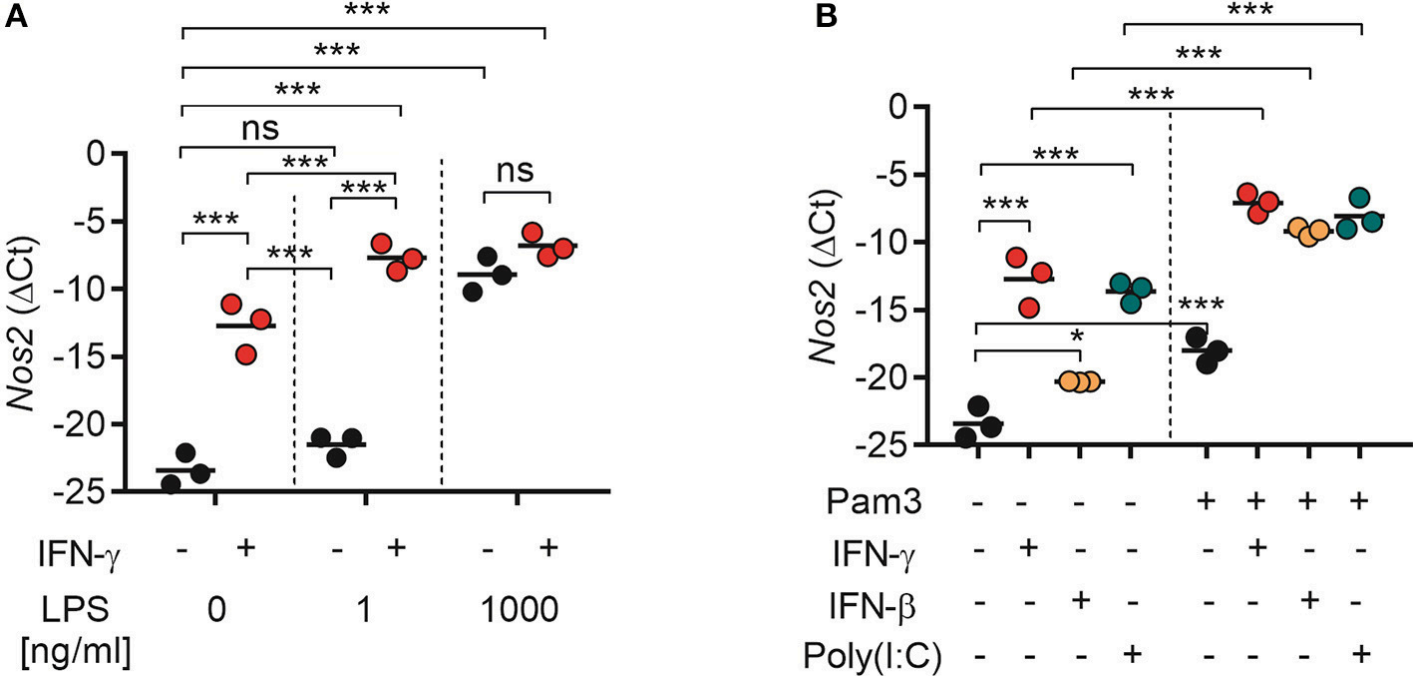
Both type I and II IFNs synergize with TLR agonists for induction of *Nos2* gene expression. BMDMs (4.5 × 10^5^) were stimulated for 24 h. Total RNA was reverse transcribed into cDNA and analyzed by RT-qPCR using primers specific for mouse *Nos2* mRNA. **(A)** *Nos2* expression in BMDMs treated with IFN-γ (40 ng/mL), or LPS at the indicated concentrations, or a combination of both. **(B)** *Nos2* expression in BMDMs treated with IFN-γ (40 ng/mL), IFN-β (40 ng/mL), poly(I:C) (100 μg/mL), Pam3CSK4 (100 ng/mL), or the indicated combinations. Data are presented as means (ΔCt) of duplicates for each of the three independent experiments. Each circle in the graphs represents the mean value from one experiment. *P*-values from a two-way ANOVA multiple comparison test is displayed as follows: *p < 0.05, ***p < 0.001; ns, not significant; Pam3, Pam3CSK4.

Our results showed that TLR agonists can synergize with both type I IFNs (recombinant or endogenously induced by poly(I:C) stimulation) and type II IFN for induction of NO production by macrophages (Figures 4, 6). In accordance with previous reports, NO was found to be critical for macrophage-mediated cancer cell growth inhibition (Figure 5) (19). To clarify the underlying mechanism, we analyzed the expression of *Nos2*, which is the gene coding for iNOS, by RT-qPCR. BMDMs were treated for 24 h with TLR agonists alone, IFNs alone, or combinations of both types of ligands. We found that stimulation with IFN-γ alone induced significant *Nos2* expression in BMDMs compared with the untreated control (Figure 7A). LPS also induced significant *Nos2* expression, but only when it was used at high concentrations (1,000 ng/mL) (Figure 7A). Stimulation with IFN-γ and LPS had a synergistic effect and resulted in significant *Nos2* expression also at low LPS concentrations (1 ng/mL). IFN-γ did not further increase the expression of *Nos2* induced by high concentrations of LPS (Figure 7A).

Stimulation with Pam3CSK4, recombinant IFN-β or poly(I:C) alone induced significant *Nos2* expression in BMDMs (Figure 7B). Furthermore, the *Nos2* expression induced by treatment with IFN-γ, IFN-β, or poly(I:C) was significantly increased when the ligands were combined with Pam3CSK4 (Figure 7B). The relative levels of *Nos2* expression correlated with the levels of NO induced by single or combined activation of BMDMs with TLR agonists and IFNs as observed in the previous experiments. Taken together, these results indicate that activation of BMDMs with TLR agonists and type I or type II IFNs results in downstream signaling which converge to the induction of *Nos2* gene expression.

### Encapsulation of poly(I:C) in nanoparticles potentiates the synergistic interaction with Pam3CSK4 for inducing antitumor macrophages up to 100-fold

**Figure 8:**
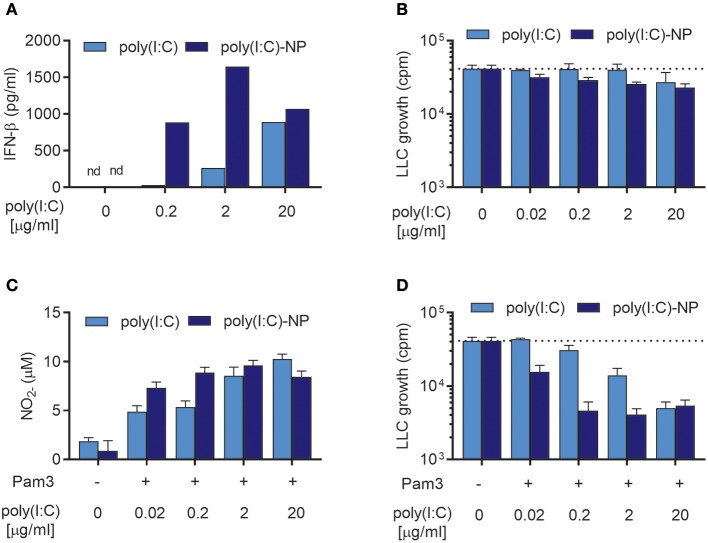
Encapsulation of poly(I:C) into nanoparticles (poly(I:C)-NPs) potentiates the synergy of combined stimulation with Pam3CSK4 and poly(I:C) in the induction of antitumor BMDMs. **(A)** Production of IFN-β by BMDMs (6 × 10^4^ in 200 μL) stimulated with the indicated concentrations of poly(I:C) either in soluble form or encapsulated in nanoparticles (poly(I:C)-NPs) for 24 h. **(B)** Growth inhibition of LLC-cells (3 × 10^3^) in co-culture with BMDMs (6 × 10^4^), stimulated for 24 h with the indicated concentrations of either soluble poly(I:C) or poly(I:C)-NPs. **(C)** Production of NO by BMDMs (6 × 10^4^) treated for 24 h with the indicated concentrations of either soluble poly(I:C) or poly(I:C)-NPs alone or in combination with 100 ng/mL Pam3CSK4. **(D)** Growth inhibition of LLC cells in co-culture with BMDMs, stimulated as described in **(C)**. Data represent the means ± SD of triplicate wells of one representative experiment out of two. nd, not detectable; Pam3, Pam3CSK4.

We have previously shown that the potency of poly(I:C) can be enhanced manifold by encapsulation into chitosan-based nanoparticles (poly(I:C)-NPs) (21). Encapsulation can protect poly(I:C) against enzymatic degradation and enhance the uptake of poly(I:C) by macrophages (21). When tested in combination with TLR ligands such as Lipomannan or BCG bacteria, poly(I:C)-NPs were up to 100-fold more potent than soluble poly(I:C) at activating BMDMs in respect to production of NO and pro-inflammatory cytokines (21). We therefore compared the ability of poly(I:C)-NPs to that of soluble poly(I:C) to induce antitumor BMDMs in the presence of Pam3CSK4. BMDMs stimulated with poly(I:C)-NPs for 24 h produced higher levels of IFN-β compared to BMDMs stimulated with the same concentrations of poly(I:C) in soluble form (Figure 8A). BMDMs stimulated with poly(I:C)-NPs alone displayed a modest growth inhibitory effect on LLC cells, possibly through the potent induction of IFN-β production (Figure 8B).

Both soluble poly(I:C) and poly(I:C)-NPs synergized with Pam3CSK4 resulting in dose-dependent NO production (Figure 8C) but failed to induce NO production by BMDMs when used alone (Supplementary Figure 2). At low concentrations of poly(I:C) there was a tendency toward higher NO production by poly(I:C)-NP compared to soluble poly(I:C) (Figure 8C). This improved potency of poly(I:C)-NP was more apparent in the growth inhibition assay, however, when combined stimulation with Pam3CSK4 of the BMDMs was necessary. Whereas, maximal inhibition of LLC growth by BMDMs was achieved already at 0.2 μg/mL of poly(I:C)-NP, a concentration of 20 μg/mL was required to achieve the same effect for soluble poly(I:C) (Figure 8D). Taken together, encapsulation of poly(I:C) into poly(I:C)-NPs increased the ability of poly(I:C) to induce IFN-β production by BMDMs and to synergize with Pam3CSK4 in inducing antitumor macrophages. In consequence, the concentration of poly(I:C) required to induce almost complete growth inhibition of LLC cells was reduced from 20 to 0.2 μg/ml by using poly(I:C)-NPs as compared to soluble poly(I:C).

## Discussion

In this study, we describe a common function of type I and type II IFNs in the activation of antitumor macrophages *in vitro*. It is well-established that LPS, a commonly used agonist for *in vitro* activation of macrophages, induces production of type I IFN through triggering of the TRIF-dependent signaling pathway (24, 25). Autocrine type I IFN signaling through STAT1 was previously found to be critical for LPS-induced iNOS expression by mouse macrophages *in vitro* (27). An early study by Vadiveloo et al. showed that *Ifnar1*^−/−^ BMDMs did not produce NO in response to LPS alone. However, these macrophages responded strongly to LPS in combination with recombinant IFN-γ, and produced NO (40). We and others have previously shown that macrophage-mediated cancer cell growth inhibition depends on NO production by mouse macrophages (19, 41, 42). Here, we found that the ability of LPS to induce macrophage-mediated cancer cell growth inhibition in the absence of IFN-γ depended on the induction of autocrine type I IFN signaling, which in turn was essential for the induction of NO production by BMDMs. These results are in agreement with the study by Vadiveloo et al. (40), as is the finding that in the presence of IFN-γ, NO production, and antitumor activation of BMDMs by LPS did not depend on autocrine type I IFN signaling. In addition, we found that the type I IFN, IFN-β had a direct inhibitory effect on the growth of the LLC cancer cell line, whereas IFN-γ or IFN-α type A did not reduce LLC growth. The observed growth inhibitory effect of IFN-β on LLC cells is in agreement with previous studies (38, 43) and can explain the partially retained ability of LPS-stimulated *Ifnar1*^−/−^ BMDMs to inhibit cancer cell growth, despite their inability to respond to type I IFNs and to produce NO.

A study from 1987 by Koestler et al. demonstrated that LPS can be combined with IFN-γ, or alternatively with IFN-α/β for induction of antitumor macrophages (44). However, to the best of our knowledge, no other study has investigated the ability of type I IFNs to activate macrophages to an antitumor phenotype in combination with other TLR agonists. We therefore tested whether recombinant IFN-β could synergize with two other TLR agonists, Pam3CSK4 (TLR1/2) and CL264 (TLR7) in inducing NO production and macrophage-mediated cancer cell growth inhibition by BMDMs. Both TLR agonists, by themselves, failed to induce production of IFN-α and IFN-β, and the macrophages required a second stimulus in form of IFN-γ to achieve an antitumor phenotype. Recombinant IFN-β and IFN-α type A were able to synergize with both of the TLR agonists for induction of NO production and macrophage-mediated inhibition of cancer cell growth. Both IFN-α and IFN-β were found to be able to replace IFN-γ as a second signal for antitumor macrophage activation, even though the type I IFNs were less effective than IFN-γ when compared at the same concentrations. However, BMDMs activated with TLR agonists in combination with any of the IFNs inhibited growth of LLC cells through a NO-dependent mechanism.

The synergistic effect of LPS and IFN-γ on NO production by macrophages has been well-described, and investigations into the underlying mechanisms have revealed that the two factors synergize to induce transcription of *Nos2* (45)*. Nos2* is encoding the enzyme iNOS which is responsible for NO production by macrophages (46). By RT-qPCR analysis of macrophage total RNA, we found that both IFN-γ and IFN-β (exogenous or endogenous) synergized with TLR signaling for induction of *Nos2* mRNA. Previously, several factors have been described to contribute to the synergistic effect of TLR and IFN signaling on iNOS expression. The promoter regions of iNOS contain binding sites for both NF-κB, IFN-γ-activating sites (GASs) and IFN-stimulated responsive elements (ISREs) (47, 48). Phosphorylated STAT1, which is induced by both type I and type II IFNs, has been shown to be able to bind GASs and ISREs and induce iNOS expression (49). Furthermore, TLR-induced activation of NF-κB and IFN-induced STAT1 and STAT2 activation has been reported to result in transcription of the *Nos2* gene in macrophages through RNA polymerase II recruitment to the *Nos2* promoter (50).

*In vivo*, IFN-γ has been shown to play a key role in mediating tumor surveillance and tumoricidal activity of the immune system by exerting direct antitumor effects such as inhibiting angiogenesis and cancer cell growth, sensitizing cancer cells to apoptosis or by activating tumoricidal activity in T cells and macrophages (51). Despite these important functions in anti-tumor immune responses, clinical use of IFN-γ for cancer treatment in general and for antitumor activation of TAMs in particular has so far been hampered by significant dose-limiting toxicities and the complex pleiotropic effects of IFN-γ on a large number of different cell types (52). These challenges could potentially be met by more targeted therapies, such as IFN-γ gene delivery to tumors by oncolytic viruses (39). The finding that type I IFNs, in a similar way as IFN-γ, can synergize with TLR agonists to induce antitumor macrophages opens up for further alternative treatment strategies targeting TAMs. Induction of endogenous type I IFN production by the TLR3 agonist poly(I:C) was previously utilized as a strategy to improve the immunogenicity of BCG, either by coating live BCG mycobacteria with poly(I:C) (53) or by combined activation with BCG and poly(I:C)-encapsulating chitosan nanoparticles (21). In both studies, poly(I:C) was shown to synergize with the TLR2 agonist BCG for production of NO and proinflammatory cytokines by macrophages, and this synergy was dependent on activation of the TRIF-dependent pathway and autocrine type I IFN signaling (21). Interestingly, Cardif-KO macrophages showed no difference to WT macrophages in induction of NO, proinflammatory cytokine or IFN-β production in response to activation with the combination of BCG and poly(I:C)-encapsulating nanoparticles (21). This demonstrates that the effect of poly(I:C) is mediated through activation of the TLR pathway rather than the Rig-1/Mda5-Cardif pathway. Multiple other studies have described similar synergy between MyD88-dependent and TRIF-dependent TLR agonists in immune cell cytokine production (54$#x02013;^57^). Poly(I:C) is currently being investigated as a potential cancer vaccine adjuvant, with at least two poly(I:C)-based drugs in clinical development (Hiltonol^®;^ and Ampligen^®;^) (58). In our study, we show that poly(I:C) induces a robust type I IFN production by BMDMs, which allows poly(I:C) to synergize with Pam3CSK4 for induction of antitumor macrophages. Furthermore, the potency of poly(I:C) could be improved ~100-fold by encapsulation into nanoparticles that we have recently developed (21). Nanoparticles are able to protect poly(I:C) against enzymatic degradation and improve uptake of poly(I:C) by macrophages, resulting in improved stability and potency of poly(I:C)(21, 59). The increased efficacy of poly(I:C)-NPs, compared to soluble poly(I:C) in inducing antitumor macrophages is likely due to a combination of (i) a modest increase in NO production and (ii) a strong increase in production of IFN-β which mediates direct and indirect antitumor effects. Further studies are required to verify that Pam3CSK4 and soluble or nanoparticle-encapsulated poly(I:C) can target and activate tumor-associated macrophages *in vivo*.

**Figure 9:**
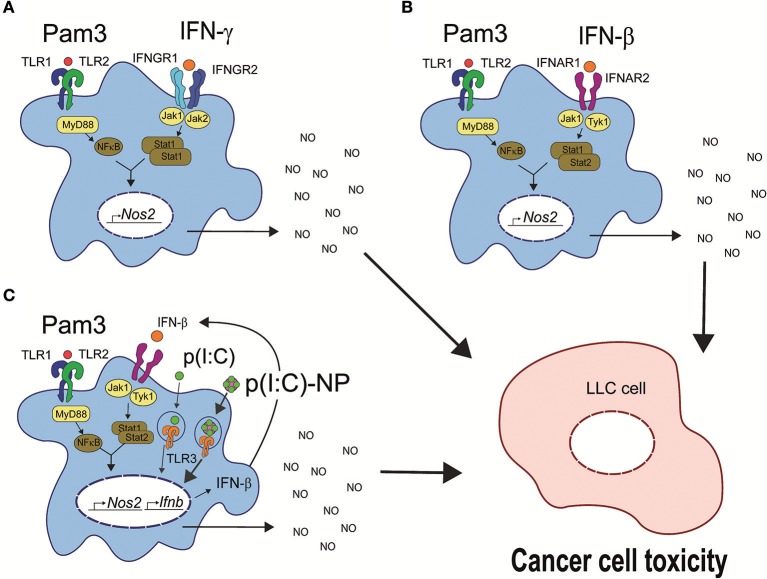
Three possible pathways for induction of antitumor macrophages. **(A)** TLR agonists such as LPS or Pam3CSK4 synergize with IFN-γ for induction of antitumor macrophages. TLR signaling through MyD88 leads to activation of NF-κB, and IFN-γ signaling through the tyrosine kinases Jak1 and Jak2 leads to STAT1 homodimer formation (61). The two pathways synergize to induce transcription of *Nos2* and NO production resulting in cancer cell growth inhibition and cell toxicity. **(B)** Synergy between a TLR agonist such as Pam3CSK4 with recombinant IFN-β for induction of antitumor macrophages. IFN-β signals through the tyrosine kinases Jak1 and Tyk2 resulting in STAT1/STAT2 heterodimer formation (62), which synergize with TLR-induced NF-κB for induction of *Nos2* gene transcription similar to **(A)**. **(C)** The TLR agonists Pam3CSK4 and poly(I:C) synergize through endogenous type I IFN production for induction of antitumor macrophages. Poly(I:C) signals through TLR3 and induces endogenous IFN-β production, which mediates the second signal and synergizes with TLR signaling as described for exogenous IFN-β in **(B)**. Pam3, Pam3CSK4.

Our results suggest that macrophages can be activated to an antitumor phenotype, characterized by high iNOS expression, NO production, and the ability to inhibit cancer cell growth by three different scenarios summarized in Figure 9. The possible therapeutic potential of activating antitumor macrophages in these ways will need to be explored *in vivo*. Finally, there are differences between mouse and human macrophages in the induction of iNOS expression as well as disagreement regarding the importance of this pathway for macrophage functions in humans (60), and it remains to be investigated whether human macrophages can be activated by similar combinations of TLR agonists and IFNs.

## Author contributions

EM contributed to the conception and design of the experiments. Performed experimental work, prepared all figures and wrote the manuscript. MS contributed to the conception and design of the experiments in collaboration with EM, prepared poly(I:C)- encapsulating nanoparticles, performed experimental work, analyzed results, and contributed to writing the manuscript. PC performed experimental work and contributed to the writing of the manuscript. AL performed experimental work and analyzed results. AA performed early, preliminary experimental work. IØ provided supervision and experimental help, discussed the results, and contributed to writing the final version of the manuscript. AC designed, supervised, and evaluated the experiments and contributed to writing the manuscript. All authors read and approved the final version of the manuscript.

### Conflict of interest statement

The authors declare that the research was conducted in the absence of any commercial or financial relationships that could be construed as a potential conflict of interest.

## Abbreviations

BCG: bacille Calmette-Guérin
BMDM: bone marrow-derived macrophage
CCK-8: cell counting kit-8
cpm: counts per minute
FBS: fetal bovine serum
IFN: interferón
IFNAR1: interferon alpha/beta receptor 1
iNOS: inducible nitric oxide synthase
LLC: Lewis lung carcinoma
LPS: lipopolysaccharide
M-CSF: macrophage colony-stimulating factor
MyD88: myeloid differentiation primary response 88
NF-κB: nuclear factor kappa-light-chain-enhancer of activated B cells
NO: nitric oxide
${\text{NO}}_{2}^{-}$: nitrite
Pam3CSK4: tripalmitoylated lipopeptide CysSerLys4
PBS: phosphate buffered saline
Poly(I:C): polyinosinic:polycytidylic acid
poly(I:C)-NP: poly(I:C)-encapsulating nanoparticles
RT-qPCR: real-time quantitative polymerase chain reaction
TAM: tumor-associated macrophage
TLR: toll-like receptor
TRIF: toll/interleukin-1 receptor homologous region (TIR)-domain-containing adapter-inducing interfere on-β.
