# Supplementary material for: Both Type I and Type II Interferons Can Activate Antitumor M1 Macrophages When Combined With TLR Stimulation
Source: Front Immunol. 2018 Nov 2;9:2520. doi: 10.3389/fimmu.2018.02520 (PMC6224375; doi:10.3389/fimmu.2018.02520)
Supplement: Supplementary file 1 [file Data_Sheet_1.pdf]

Supporting information

# Both Type I and Type II Interferons Can Activate Antitumor M1 Macrophages When Combined With TLR Stimulation

Elisabeth Müller[1,2*], Martin Speth[2], Panagiotis F. Christopoulos[1], Anna Lunde[1], Ajna Avdagic[1], Inger Øynebråten[1], and Alexandre Corthay[1*]

Affiliations:
[1]Tumor Immunology Lab, Department of Pathology, Rikshospitalet, Oslo University Hospital, University of Oslo, Oslo, Norway, [2]Department of Biosciences, University of Oslo, Oslo, Norway

***Correspondence:**
Elisabeth Müller - elisabeth.mueller@ibv.uio.no;
Alexandre Corthay - alexandre.corthay@ibv.uio.no

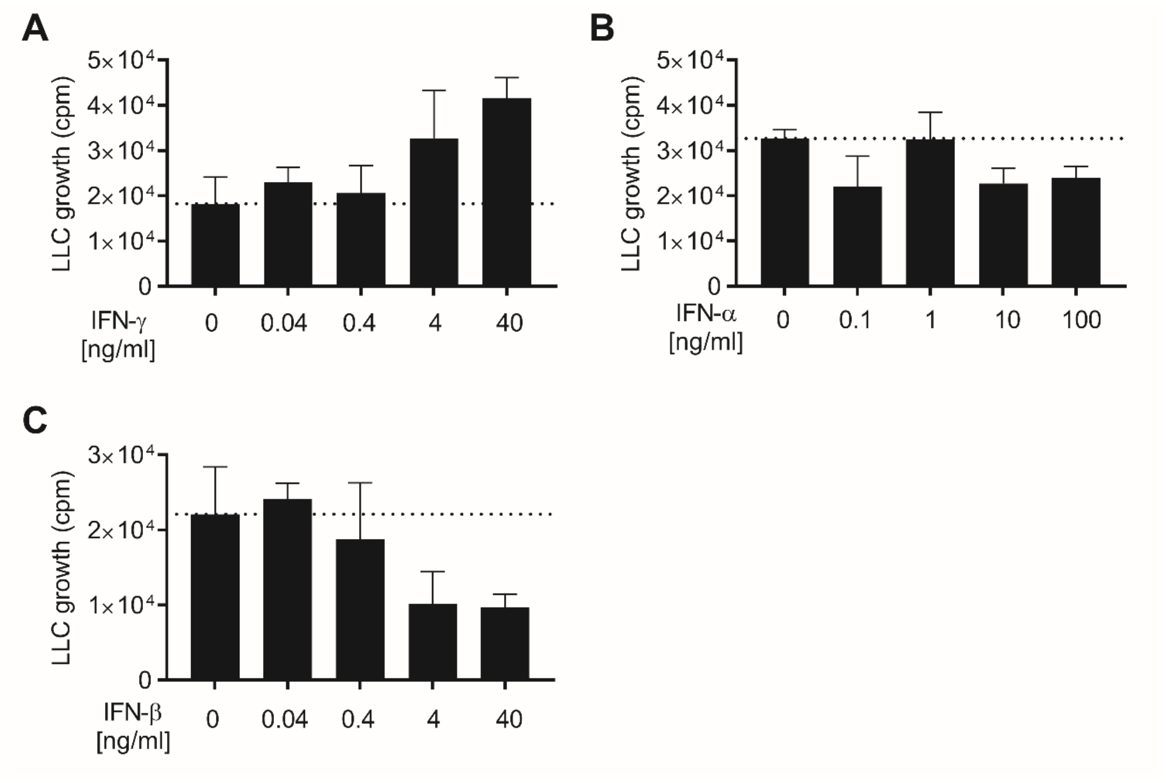

Supplementary Figure 1.

LLC cell growth after 42 h of incubation with different concentrations of (**A**) recombinant IFN-γ, (**B**) IFN-β or (**C**) IFN-α type A. LLC cells ($3x10^3$ per well) were seeded out and left untreated or incubated with the indicated IFNs for 24 h. Then, radiolabeled thymidine was added to the cultures and LLC cells were incubated for another 18 h. Finally, LLC were harvested and thymidine incorporation was analyzed as a measure of LLC cell growth. Results are expressed as counts per minute (cpm). Data are presented as means ± SD of triplicate wells of one representative experiment out of three.

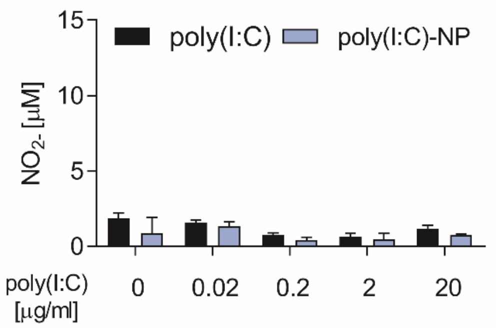

Supplementary Figure 2.

Induction of NO production as measured indirectly as NO_2^- by the Griess assay upon activation of BMDMs ($6x10^4$ per well) with soluble poly(I:C) or poly(I:C) encapsulated in nanoparticles, poly(I:C)-NP, for 24 h. Data are presented as means ± SD of triplicate wells of one representative experiment out of two.
